# Bone Morphology and Strength in the Mid-Diaphysis of the Humerus and Metacarpus in Dairy Calves Prior to Weaning

**DOI:** 10.3390/ani10081422

**Published:** 2020-08-14

**Authors:** Michaela Gibson, Keren Dittmer, Rebecca Hickson, Penny Back, Chris Rogers

**Affiliations:** 1 School of Agriculture and Environmental Sciences, Massey University, Palmerston North Private Bag 11-222, New Zealand; R.Hickson@massey.ac.nz (R.H.); P.J.Back@massey.ac.nz (P.B.); C.W.Rogers@massey.ac.nz (C.R.); 2 School of Veterinary Sciences, Massey University, Palmerston North Private Bag 11-222, New Zealand; K.E.Dittmer@massey.ac.nz

**Keywords:** bone, fracture, humerus, metacarpus, dairy calf, bone strength

## Abstract

**Simple Summary:**

Calf growth and nutrition has been thoroughly researched in relation to future milk production. The effect of nutrition on growth has been overlooked, however, and the emergence of humeral fractures in first lactation heifers has driven the need for further research. The aim of this study was to understand the relationship of calf growth with bone measures such as size and density prior to weaning. Liveweight, height, body length, girth and leg length were measured at one, six and twelve weeks of age. At these intervals, the middle of the metacarpus was also scanned using a CT scanner. At the six and twelve weeks measures, a subset of calves were euthanised and the humerus was collected and scanned in the middle of the bone. Differences in growth between the metacarpus and humerus were observed over time. Liveweight was the main driver of the majority of bone measures in both the metacarpus and humerus. The strong relationship between weight and bone shows the importance of adequate preweaning nutrition to ensure adequate future bone growth.

**Abstract:**

Calf growth rate in relation to future milk production has been thoroughly studied; however, the observation of growth arrest lines in bones from heifers with humeral fractures has highlighted the need to understand bone growth in relation to calf growth. The aim of this study was to describe the relationship of peripheral quantitative computed tomography (pQCT)-derived measures of bone strength and morphology with gross measurements of size and growth in pre-weaning dairy calves. Liveweight, height, body length, girth and leg length were measured at one, six and twelve weeks of age. At these intervals, the mid-diaphysis of the metacarpus was also scanned in the live animal using pQCT. At six and twelve weeks old, a subset of calves were euthanised and the humerus was collected and scanned at the mid-diaphysis using pQCT. Differences in growth patterns were observed between the metacarpus and humerus over time. Weight was the best predictor for measures of periosteal circumference and stress strain index (R^2^ = 0.49–0.58) in the metacarpus, and also the best predictor for measures of stress strain index at all ages in the humerus (R^2^ = 0.94). The strong relationship with weight and bone measures emphasises the need for adequate preweaning nutrition for future bone growth.

## 1. Introduction

The association of growth and future production in dairy cattle has been well described within the literature [1,2]. However, the effect of growth and environment on subsequent bone development in dairy heifers has often been overlooked. Humeral fractures in first lactation dairy heifers were first reported in 2008 and represent a significant economic and welfare issue for the New Zealand dairy industry, with an estimated within-herd incidence of 4–25%, and approximately 5000 dairy replacements each year may be affected [3]. A case-control study of humeral fractures showed a reduction in cortical thickness and osteoporosis. While the risk factors for humeral fracture are multifactorial, a major contributor is believed to be poor nutrition early in a heifer’s life [3]. However, there is currently no published literature to inform on the timing and severity of nutrition restriction required to have an effect.

Bone growth and development has been thoroughly described, particularly in relation to osteoporosis, in humans and animal (murine) models. Historically, particularly in human studies, data have been obtained using dual-energy X-ray absorptiometry (DEXA), which had been considered the gold standard of non-invasive measurements. However, a limitation of DEXA is the inability to distinguish between changes in cortical and cancellous bone due to scanning a three-dimensional model (bone) in two dimensions. Using DEXA, bone mass can be measured, but changes in cancellous bone and more importantly cortical bone cannot be quantified [4]. In contrast, peripheral quantitative computative tomography (pQCT) provides a non-invasive technique of determining bone parameters but is able to describe not just bone mass and density, but also measures of bone geometry, and thus, calculation of precise measures of bone strength and resistance to fracture [5].

There is limited published literature describing the effect of sex and other factors on bone growth and development in livestock, with much of the published literature focusing on horses [6,7]. There are limited data on bone growth and development in cattle; however, the majority of these have been collected using DEXA, preventing description of changes in bone geometry and microarchitecture [8]. Therefore, the aim of this study was to describe the relationship of pQCT-derived measures of bone strength and morphology within two different bones, in the proximal and distal limb, with gross measurements of size and growth.

## 2. Materials and Methods

This report details the prospective observational study with serial and terminal sampling to ensure measurement of bone parameters at the mid-diaphysis of two bones in the proximal and distal limb on three separate occasions during pre-weaning growth. Measures of frame size and weight were taken to allow for comparison of size and bone parameters.

### 2.1. Animals and Management

The cohort consisted of 21 female Friesian–Jersey crossbred calves born at Massey University’s Dairy 1 farm during the 2016 spring calving season. Calves were fed ad libitum colostrum from birth to three days old. At four days of age, calves were assigned into two feed groups and offered either 10 L (*n* = 9) or 5 L (*n* = 12) of milk per day, split over two feeds (am and pm). From birth, calves were housed in a shed, and at approximately three weeks old, all calves were managed at pasture, excluding a subset of six calves from the 5 L cohort that remained in the shed with the intention to restrict exercise. The six calves that remained in the rearing shed were offered access to a balanced ration of meal (Sharpes Pellets, Commercial mix, 18% crude protein) at a rate that provided equivalent estimated daily digestible energy (DE) and nutrients to those of the calves with access to pasture.

### 2.2. Sampling and Measurements

Calves were measured at birth and at time of pQCT scanning for live weight and the following stature measures: wither height, girth, leg length (from ground to elbow of the right forelimb) and wither-rump length (stature measures).

In cattle, the third and fourth metacarpus are fused and are subsequently referred to as the metacarpus within the text. The metacarpus (MC3/MC4) was scanned at one (scan one), six (scan two) and twelve (scan three) weeks of age. The metacarpus of the right limb was scanned, whilst calves were in lateral recumbency and anaesthesia was induced with either 6 mg/kg followed by 0.3 mg/kg/min during anaesthesia of propofol (Fresofol 1% MCT/LCT) or 2 mg/kg followed by 0.2 mg/kg/min during anaesthesia of Alfaxalone (Alfaxan multidose 10 mg/mL).

Of the 21 animals, eight were euthanised at the six weeks scan (three from 5 L restricted exercise, three from 5 L unrestricted exercise and two from 10 L) and eight animals at the twelve weeks scan (three from 5 L restricted exercise, two from 5 L unrestricted exercise and three from 10 L). One calf from the 10 L group was euthanised after the one week scan and one calf from the 5 L non-restricted exercise group after the six weeks scan due to ill-thrift not related to the current study. This left three live calves from the 10 L group after the twelve weeks scan that were returned to the herd for a future study. At euthanasia, the humerus and metacarpus were dissected out and cleaned of muscle mass, double-wrapped in plastic and stored frozen (−20 °C freezer) until subsequent pQCT scanning. All procedures related to the management and use of the animals during the experiment were approved by the Massey University Animals Ethics Committee (MUAEC 19/81).

### 2.3. Sampling Techniques

All pQCT scanning was carried out using an XCT 2000 peripheral quantitative computed tomography machine (Stratec Medical). For each bone, a 2 mm slice at the mid-diaphysis was obtained with a voxel size of 0.3 mm^3^. In the metacarpus, the mid-diaphysis was defined as 50% of the total bone length using the lateral aspect of the lateral condyle of the fourth metacarpal (MC4) and the proximal aspect of the lateral MC4. In the humerus, the mid-diaphysis was defined as 50% of total bone length, measured from the most distal aspect of the trochlea, to the proximal end of the humeral head at the lateral aspect.

Within the manufacturers software, voxels > 710 mg/cm^3^ were assigned as “cortical” bone. Data derived from the scan included measures of total bone content, cortical and subcortical content, cortical and subcortical density, trabecular content, trabecular density, total area, trabecular area, cortical and subcortical area, cortical content, cortical density, cortical area, cortical thickness, periosteal circumference, endosteal circumference and stress strain index (SSI). The stress strain index is the ability to withstand bending from lateral, dorso-palmar and torsional forces and is calculated by incorporating the index of material stiffness (bone mineral density) and bone geometry (cross-sectional moment of inertia) [9]. These are referred to as bone parameters in the statistical models.

### 2.4. Statistical Analysis

Statistical analysis was conducted using SAS version 9.4 (SAS Institute Inc., Cary, NC, USA). Bone parameters and stature measures were analysed using a general linear model that included the fixed effect of age at measurement. Rearing treatment (milk allowance and exercise) was tested in this model but was removed because it failed to reach significance (*p* > 0.05) for any parameter considered. Similarly, change in bone parameters and stature measures were analysed using a general linear model that included the fixed effect of period (first to second scan versus second to third scan). Bone parameters were regressed on body weight on day of measurement to calculate the regression coefficient for body weight at each measurement age. Bone parameters were analysed using a multivariate regression with stepwise selection, which considered stature measures as predictors of each bone parameter within measurement event. The threshold for inclusion in the multivariate regression model was *p* < 0.05.

## 3. Results

The calves had a mean birth weight of 35.2 ± 0.9 kg with a range of 29.0–42.5 kg. At the twelve weeks scan, the age at which calves would have been weaned, they weighed on average 101.9 ± 2.3 kg. At the time of each pQCT scan, stature measures had increased significantly from the previous measure (Table 1, *p* < 0.05).

At the mid-diaphysis site of the metacarpus, there were significant increases with age at scanning (*p* < 0.05) in all bone parameters measured with the exception of endosteal circumference, which did not change between scans (Table 1). There were significant increases in most of the bone parameters of the humerus at six and twelve weeks of age (*p* > 0.05), excluding cortical density. Mean bone measures were greater in the humerus than the metacarpus at each age, including stress strain index which at 12 weeks was 3398.9 ± 146.4 mm^3^, which was approximately twice that calculated for the metacarpus (1431.4 ± 49.4 mm^3^). The primary contributors to the increase in stress train index during growth differed between the two bones. In the metacarpus, between the six to twelve weeks scan, there was an 11% increase in periosteal circumference and 30% increase in cortical thickness, which resulted in a 49% increase in stress strain index. In contrast, in the humerus, a 22% increase in periosteal circumference in association with a similar 27% increase in cortical thickness resulted in a 91% increase in the stress strain index.

At the mid-diaphysis site of the metacarpus, increases in total area and periosteal circumference were positively associated with bodyweight (Table 2). In contrast, endosteal circumference at twelve weeks was better explained by wither-rump length and height. In the regression model for cortical content in the metacarpus at six weeks, weight was the best stature measure to explain increases in content. However, at one and twelve weeks, girth slightly improved the fit of the regression model, compared to the use of liveweight.

In the humerus, measures of cortical/subcortical density were positively associated with wither-rump length (Table 2). When using a stepwise selection model, girth was a major predictor of total size and area, but there was only moderate improvement above using only weight in the model. Total content, cortical thickness, cortical area, cortical content, cortical/subcortical area, cortical/subcortical content and stress strain index were positively associated with weight (R^2^ = 0.73 − 0.94); however, cortical thickness was also negatively associated with height. Cortical density was not significantly affected by any stature measure (*p* < 0.05).

## 4. Discussion

The calves were reared on a commercial dairy farm using standard New Zealand farming practices. At twelve weeks of age, the average weight of 102 kg aligned with the industry standards of 90 kg at 8–12 weeks of age for a Holstein–Friesian–Jersey crossbred heifer calf [10].

Differences in the pattern of growth between the metacarpus and humerus were observed that reflected the relative growth of the two bones during the study period and the differences in mechanical loading of the bones due to bodyweight and muscular strain. Endosteal circumference did not increase in the metacarpal but periosteal circumference increased, demonstrating that cortical bone (>710 mg/cm^3^) was being deposited on the outside of the bone resulting in increased cortical thickness, bone area/size and, therefore, bone strength. However, in the humerus, endosteal circumference increased proportionally with periosteal circumference. This appositional growth is a highly efficient mechanism to increase bone strength (SSI) during growth, with minimal increases in bone mass, and thus, only a marginal increase in the resources required for bone growth.

Mechanical loading affects bone structure in the form of changing its mass and architecture of bone to optimise (i.e., minimise) the effective strain rate. The different strains applied to the two bones during growth, and the tissue (structural) responses observed, reflected their anatomical position and relative growth potential. There was a consistent association of weight with bone parameter measures in the humerus, which contrasted with the limited association (R^2^) identified with bone parameter measurements in the metacarpus. The difference in associations of stature measures with bone can be explained by the anatomical location of the two bones (proximal vs. distal), which generated very different patterns of strain, differing in both the magnitude and direction of the strain, placed on the two bones [11,12].

The greater SSI of the humerus mid-diaphysis implies that at this site, the humerus is subjected to greater force than the metacarpus. The shape of the humerus is derived by the direction and size of the muscle forces exerted on the bone as additional bone is placed on regions that have the largest bending strain [13]. Finite element analysis of the humerus indicates that first force (primary and majority of strain) on the humerus is torsion, attributed to combined force of the supraspinatus, infraspinatus, pectoralis and subscapularis muscles. The second force can be attributed to the triceps brachialis muscle. The third and final force group considered was force placed on the humerus attributed to body weight [12]. In contrast, the loading on the metacarpus in cattle is effectively loading under stance (compression) and low strain rate locomotion (i.e., walking) [11]. Girth was highly correlated with weight, so the slight improvement in model fit with the inclusion of girth may be due to girth providing an indication of frame size and, therefore, an indirect measure of thoracic growth and muscle force.

During growth, in both the metacarpus and humerus, increases in strength (SSI) were obtained by increases in circumference, with only minimal changes in cortical bone density. Increases in bone strength are also likely to be a result of changes in bone microarchitecture as primary bone is replaced with lamellar bone tissue forming osteons [14]. Bone mineralisation is much slower, so minimal changes in density would occur and the changes in architecture are unable to be detected at a pQCT level. Biologically, the first approach to increase bone strength is to increase bone size (circumference), and only after exposure to high strain rates (e.g., galloping) is there an increase in bone density. In young horses, significant increases in density at the mid-diaphysis were only observed after the introduction of intensive exercise, such as galloping [7,15]. In comparison, calves, which are not a cursorial species, do not have the same predisposition to run at high speed and are therefore unlikely to subject the distal limb to the high strain rates observed in galloping horses. Even when calves were run on a concrete path, this failed to induce sufficient high strain rates to increase bone density, with the primary response being an alteration in bone circumference and focal response in morphology to the strain (deposition of bone on the dorsal surface) [8]. The results from the current study agree with those of Hiney, et al. [16] that calves on a pasture-based system will not reach the critical loading threshold to induce an increase in cortical density and no differences in cortical density between calves on restricted and unrestricted exercise would be expected.

The rate at which the humerus increased in strength (SSI) was higher than what is observed in the metacarpal bone, reflecting that the majority of bone growth in these animals was in the proximal rather than distal limb. This rapid and sustained growth in the humerus, in association with increases in periosteal circumference but not cortical thickness, may predispose the humerus to pre-pubertal growth perturbations due to malnutrition or illness. Pre-pubertal growth perturbations may affect the ability of the humerus reach peak bone mass and therefore increase the risk of humeral fracture in severe cases. This highlights the need for adequate growth prior to weaning and puberty, to not only allow animals to maximise production performance but to ensure peak bone mass can be achieved.

It is possible that the effect of pre-pubertal malnutrition (i.e., feed restriction) on bone development does not become obvious, or quantifiable, without histological examination, until later in life. The effect of diet, in this study, did not influence stature or any of the bone parameters so was not included within the model. It is possible that the lack of effect of diet on bone parameters was due to the low sample number of calves in each treatment group. However, it is likely that if an effect of diet was to occur, it would occur later in life as observed by Moallem, et al. [17]. Therefore, the primary effect of diet (differing nutrition planes) may be alteration of the developmental pathway, which may have been observed if scans had been repeated at an older age after the pre-weaning growth period. Support for this hypothesis can be derived from observations of the effect of early activity (including pre-weaning high strain rate exercise) on bone development up to post-puberty in foals. The provision of additional canter exercise (30% higher workload than controls at pasture) did not have a significant quantifiable effect on bone size and strength until the horses were 12 months old. It is likely that changes were occurring to the developmental potential and microarchitecture of bone, but changes in gross morphology of the bone were not observable until later in age [9]. It is possible that a similar effect occurred in the calves in the current study, where developmental programming may have been occurring between the dietary treatments. If the effect of diet on weight becomes significant at a later age as proposed by Moallem, Werner, Lehrer, Zachut, Livshitz, Yakoby and Shamay [17]), then the effect of diet may have become quantifiable using pQCT at a later age.

## 5. Conclusions

The current study demonstrated that weight and, to a lesser degree, girth are the main explanatory factors of bone development and strength in calves prior to weaning. The strategy to increase resistance to bending strain and torsion (SSI) differed between the humerus and the metacarpus. The rapid growth of the humerus during this pre-weaning period and the increases in periosteal circumference and cortical thickness provide an explanation of why the humerus may be more susceptible to growth checks. Changes in cortical thickness in the metacarpal are likely to be attributed by factors not measured in the current study. The strong association of bone development with bodyweight indicates that in a practical setting, bodyweight and average daily gain provide a good proxy measure for bone development and attainment of bone mass in pre-weaning dairy heifers. If the relationship between bone strength and weight is disrupted, bone may not be able to reach adequate strength to withstand forces. Therefore, it can be concluded that satisfactory pre-weaning growth rates are required to maintain the relationship between weight and bone strength for future growth.

## Figures and Tables

**Table 1:** Least squares means and standard error of stature measures and peripheral quantitative computed tomography (pQCT) bone measures of the humerus and metacarpus at 1, 6 and 12 weeks of age in dairy calves.

Age	1 Week	6 Weeks	12 Weeks	*p*-Value
Stature				
*n*	21	20	11	
Liveweight (kg)	36.1 ± 1.6 ^a^	57.0 ± 1.65 ^b^	102.0 ± 2.3 ^c^	<0.001
Age (days)	6.1 ± 0.6 ^a^	38.9 ± 0.6 ^b^	84.1 ± 0.9 ^c^	<0.001
Wither-rump length (cm)	87.0 ± 1.3 ^a^	97.2 ± 1.4 ^b^	118.2 ± 1.9 ^c^	<0.001
Height (cm)	75.0 ± 0.8 ^a^	82.0 ± 0.8 ^b^	94.6 ± 1.1 ^c^	<0.001
Girth (cm)	76.8 ± 0.9 ^a^	89.8 ± 0.9 ^b^	107.4 ± 1.3 ^c^	<0.001
Leg Length (cm)	48.6 ± 0.7 ^a^	51.9 ± 0.7 ^b^	57.8 ± 1.0 ^c^	<0.001
**Metacarpus**				
*n*	21	20	11	
Bone length (mm)	160.2 ± 1.3 ^a^	168.9 ± 1.4 ^b^	181.0 ± 1.9 ^c^	<0.001
Periosteal circumference (mm)	60.6 ± 0.9 ^a^	63.5 ± 1.0^b^	70.6 ± 1.4 ^c^	<0.001
Endosteal circumference (mm)	43.6 ± 1.1	43.6 ± 1.1	45.5 ± 1.5	0.546
Total bone area (mm^2^)	293.3 ± 9.3 ^a^	321.1 ± 9.5 ^b^	397.5 ± 13.4 ^c^	<0.001
Total bone content (mg/mm)	181.2 ± 4.7 ^a^	218.7 ± 4.8 ^b^	297.5 ± 6.8 ^c^	<0.001
Cortical bone thickness (mm)	2.7 ± 0.1 ^a^	3.1 ± 0.1 ^b^	4.0 ± 0.1 ^c^	<0.001
Cortical bone area (mm^2^)	140.5 ± 3.7 ^a^	167.8 ± 3.8 ^b^	231.1 ± 5.4 ^c^	<0.001
Cortical bone content (mg/mm)	151.1 ± 4.2 ^a^	194.35 ± 4.4 ^b^	269.6 ± 6.0 ^c^	<0.001
Cortical bone density (mg/cm^3^)	1075.7 ± 5.8 ^a^	1142.9 ± 6.0 ^b^	1166.7 ± 7.4 ^c^	<0.001
Cortical/Subcortical area (mm^2^)	166.8 ± 1.0 ^a^	194.7 ± 4.1 ^b^	262.2 ± 5.8 ^c^	<0.001
Cortical/Subcortical content (mg/mm)	163.7 ± 4.5 ^a^	204.65 ± 4.6 ^b^	284.5 ± 6.5 ^c^	<0.001
Cortical/subcortical density (mg/cm^3^)	981.9 ± 5.4 ^a^	1050.1 ± 6.2 ^b^	1084.8 ± 8.8 ^c^	<0.001
Stress strain index (mm^3^)	734.1 ± 34.1 ^a^	962.35 ± 34.9 ^b^	1431.4 ± 49.4 ^c^	<0.001
**Humerus**				
*n*	0	8	8	
Periosteal circumference (mm)	−	79.4 ± 2.3 ^a^	97.0 ± 2.3 ^b^	<0.001
Endosteal circumference (mm)	−	58.9 ± 2.3 ^a^	70.7 ± 2.3 ^b^	0.003
Total bone area (mm^2^)	−	504.4 ± 32.16 ^a^	752.1 ± 3.6 ^b^	<0.001
Total bone content (mg/mm)	−	305.6 ± 15.0 ^a^	466.6 ± 15.0 ^b^	<0.001
Cortical bone thickness (mm)	−	3.3 ± 0.1 ^a^	4.2 ± 0.1 ^b^	<0.001
Cortical bone area (mm^2^)	−	226.3 ± 11.4 ^a^	350.7 ± 11.4 ^b^	<0.001
Cortical bone content (mg/mm)	−	264.4 ± 12.7 ^a^	413.4 ± 12.7 ^b^	<0.001
Cortical bone density (mg/cm^3^)	−	1169.8 ± 5.3	1179.4 ± 5.3	0.228
Cortical/subcortical area (mm^2^)	−	257.3 ± 12.8 ^a^	389.3 ± 12.8 ^b^	<0.001
Cortical/subcortical content (mg/mm)	−	279.6 ± 13.4 ^a^	432.4 ± 13.4 ^b^	<0.001
Cortical/subcortical density (mg/cm^3^)	−	1087.9 ± 5.9 ^a^	1111.1 ± 5.7 ^b^	0.015
Stress strain index (mm^3^)	−	1776.4 ± 146.4 ^a^	3398.9 ± 146.4 ^b^	<0.001

**^a, b, c^** Means with different superscript within row at each age are significantly different (*p* < 0.05).

**Table 2:** Regression equations at each scan age of the metacarpal fitting with stepwise selection using weight, girth, birth weight, wither-rump length, height and leg length as potential predictors. Regression equations for the mid-diaphyseal site of the humerus for scans at 6 and 12 weeks of age fitting with stepwise selection using weight, girth, birth weight, wither-rump length, height and leg length as potential predictors. Weight R^2^ indicates the model fit when using only weight as a predictor for each bone measure at each scan. Missing value indicates that the parameter was not significant at the *p* < 0.05 threshold.

Bone	Scan	Intercept	Weight	Girth	Wither-Rump Length	Height	Leg	Weight R^2^	StepWise R^2^
METACARPUS									
Periosteal	1	31.7 ± 6.9	0.8 ± 0.2					0.49	0.49
circumference (mm)	2	42.9 ± 5.1	0.4 ± 0.1					0.50	0.50
	3	39.0 ± 10.6	0.3 ± 0.1					0.53	0.53
Endosteal	1	11.95 ± 8.2	0.9 ± 0.2					0.45	0.45
circumference (mm)	2	27.5 ± 6.7	0.3 ± 0.1					0.26	0.26
	3	−72.1 ± 30.5			0.4 ± 0.1	0.75 ± 0.3		0.32	0.72
Total area (mm^2^)	1	20.1 ± 64.7	7.6 ± 1.8					0.50	0.50
	2	119.0 ± 50.2	3.6 ± 0.9					0.50	0.50
	3	42.0 ± 122.2	3.5 ± 1.2					0.52	0.52
Total content	1	107.8 ± 33.8	2.0 ± 0.9					0.21	0.21
(mg/mm)	2	101.4 ± 21.4	2.1 ± 0.4					0.65	0.65
	3	NS						0.28	
Cortical thickness	1	NS						0.04	
(mm)	2	NS						0.15	
	3	NS						0.00	
Cortical area	1	82.5 ± 27.4	1.6 ± 0.8					0.20	0.20
(mm^2^)	2	72.4 ± 16.7	1.7 ± 0.3					0.67	0.67
	3	NS						0.30	
Cortical Content	1	−10.4 ± 73.0		2.1 ± 1.0				0.20	0.21
(mg/mm)	2	84.5 ± 20.0	1.9 ± 0.3					0.65	0.65
	3	−82.9 ± 182.7		3.3 ± 1.7				0.27	0.32
Cortical Density	1	NS						0.00	
(mg/cm^3^)	2	NS						0.01	
	3	NS							
Cortical/subcortical	1	97.7 ± 28.9	1.9 ± 0.8					0.24	0.24
area (mm^2^)	2	87.6 ± 17.1	1.91 ± 0.3					0.71	0.71
	3	NS							
Cortical/subcortical	1	93.8 ± 29.6	1.9 ± 0.8					0.23	0.24
Content (mg/mm)	2	91.4 ± 19.9	2.0 ± 0.3					0.67	0.67
	3	NS							
Cortical/subcortical	1	NS						0.00	
Density (mg/cm^3^)	2	NS						0.00	
	3	NS							
Stress strain index	1	−33.5 ± 191.7	21.3 ± 5.3					0.47	0.47
(mm^3^)	2	117.7 ± 180.1	15.0 ± 3.1					0.58	0.58
	3	−32.1 ± 503.8	14.4 ± 4.9					0.52	0.52
**HUMERUS**									
Periosteal circumference (mm)		−1.7 ± 10.3		0.9 ± 0.1				0.82	0.84
Endosteal circumference (mm)		20 ± 15.7				1.0 ± 0.1		0.62	0.68
Total bone area (mm^2^)		−634.2 ± 150.1		13.0 ±1.7				0.82	0.83
Total bone content (mg/mm)		126.6 ± 21.3	3.4 ± 0.3					0.92	0.92
Cortical bone thickness		6.1 ± 1.8	0.03 ± 0.01			−0.06 ± 0.02		0.73	0.73
Cortical bone area (mm^2^)		89.4 ± 16.9	2.6 ± 0.2					0.92	0.92
Cortical bone content (mg/mm)		21.2 ± 17.3	1.2 ± 0.2					0.68	0.68
Cortical bone density		NS						0.00	
Cortical/subcortical area (mm^2^)		101.8 ± 18.4	3.1 ± 0.2					0.93	0.93
Cortical/subcortical content (mg/mm)		112.2 ± 19.7	3.2 ± 0.2					0.92	0.92
Cortical/subcortical density		1004.7 ± 32.7			0.9 ± 0.3			0.20	0.38
Stress strain index		−30.5 ± 191.3	34.2 ± 2.4					0.94	0.94

NS = No variables met the threshold for inclusion in the model.
